# Role of epithelial-mesenchymal transition factor SNAI1 and its targets in ovarian cancer aggressiveness

**DOI:** 10.20517/2394-4722.2023.34

**Published:** 2023-06-30

**Authors:** Tise Suzuki, Ashlyn Conant, Casey Curow, Audrey Alexander, Yevgeniya Ioffe, Juli J. Unternaehrer

**Affiliations:** 1 Division of Biochemistry, Department of Basic Sciences, Loma Linda University, Loma Linda, CA 92354, USA.; 2 University of Redlands, Department of Biology, Redlands, CA 92373, USA.; 3 Division of Natural and Mathematical Sciences, Department of Biological Sciences, California Baptist University, Riverside, CA 92504, USA.; 4 Department of Gynecology and Obstetrics, Division of Gynecologic Oncology, Loma Linda University Medical Center, Loma Linda, CA 92354, USA.; 5 Department of Gynecology and Obstetrics, Loma Linda University, Loma Linda, CA 92354, USA.

**Keywords:** Snail, ovarian cancer, epithelial-mesenchymal transition, chemoresistance, cancer stemness, cancer invasion, metastasis, cancer aggressiveness

## Abstract

Ovarian cancer remains the most lethal gynecologic malignancy in the USA. For over twenty years, epithelial-mesenchymal transition (EMT) has been characterized extensively in development and disease. The dysregulation of this process in cancer has been identified as a mechanism by which epithelial tumors become more aggressive, allowing them to survive and invade distant tissues. This occurs in part due to the increased expression of the EMT transcription factor, *SNAI1* (Snail). In the case of epithelial ovarian cancer, Snail has been shown to contribute to cancer invasion, stemness, chemoresistance, and metabolic changes. Thus, in this review, we focus on summarizing current findings on the role of EMT (specifically, factors downstream of Snail) in determining ovarian cancer aggressiveness.

## INTRODUCTION

Epithelial ovarian cancer (EOC) is the most lethal among gynecological cancers in the USA^[1]^. Within its histological subtypes, serous tumors are the most frequent, followed by endometrioid, clear cell, and mucinous^[2]^. Serous carcinomas can be further classified into two main subtypes: type I (low-grade) and type II (high-grade), with high-grade accounting for approximately 75% of all EOCs^[2]^. They differ by their development rate, precursor lesions, chromosomal stability, and gene mutations^[3–5]^. High-grade serous ovarian cancer (HGSOC) is not only the most common subtype but also the most aggressive in nature, as 80% of cases are diagnosed at stages III and IV, and patients diagnosed with distant metastasis (stage IV) exhibit approximately 30% five-year survival rates^[1,6]^. Unlike breast and cervical cancer, HGSOC lacks universal screening methods for the early diagnosis of the disease, and since most early cases are asymptomatic, many remain undetected until after they become invasive^[7]^. Furthermore, currently available treatments for advanced stages, which commonly include cytoreductive surgery and chemotherapy, have low effectiveness in eradicating the heterogeneous cell populations observed within tumors^[8]^. For this reason, over 70% of patients experience relapse, and recurrent HGSOC becomes resistant to further treatment efforts aimed at disease control via chemoresistance and other mechanisms^[8]^.

Given that HGSOC has a high propensity for metastases, a hallmark of late-stage disease is the development of peritoneal carcinomatosis. For metastasis to occur, cancer cells from the primary tumor are required to undergo significant morphological change to adapt to their transportation and deposition at a secondary site. One of the leading proposed mechanisms by which metastasis occurs is through epithelial-mesenchymal transition (EMT). Several transcription factors are known to exert control over this process, including *SNAI1, SNAI2, TWIST1, ZEB1*, and *ZEB2*^[9]^. In many cancer types, including HGSOC, the expression of the EMT transcription factor *SNAI1* (Snail) has been associated with a more aggressive disease, in which cancer cells are more metastatic, stem cell-like, resistant to chemotherapy agents, and have undergone adaptive metabolic changes^[10–14]^. Moreover, Snail expression has been identified in non-epithelial cells, such as mesenchymal cells^[15]^ and cancer-associated fibroblasts (CAFs)^[11,16,17]^, that modify the tumor microenvironment by remodeling and rebuilding the extracellular matrix via matrix protease secretion and fiber deposition, thus creating an environment favorable to cancer growth and metastasis. Therefore, in this review, our purpose is to evaluate what is known of Snail’s mechanistic role in modulating ovarian cancer aggressiveness and its potential as a target for a therapeutic approach.

## SNAIL AND EPITHELIAL-MESENCHYMAL TRANSITION

**Figure 1.**
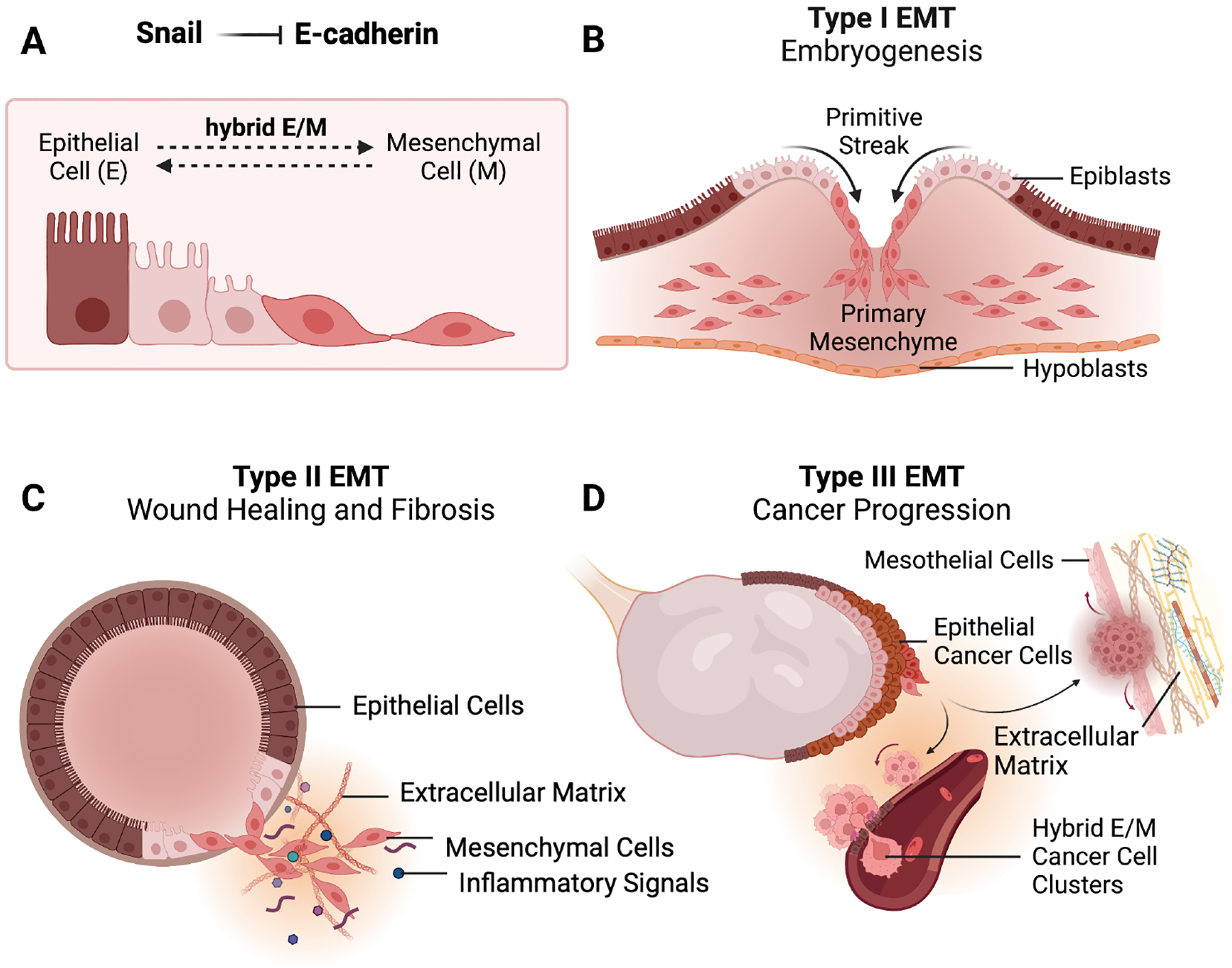
Types of epithelial-mesenchymal transition (EMT). (A) After EMT induction, transcription factors (e.g., Snail) inhibit the expression of cell adhesion molecules (e.g., cadherin 1/*CDH1*), thereby inducing morphological and phenotypic changes that prepare a cell for migration. (B) During development, type I EMT and its reverse process, MET, must occur in multiple steps for the formation of different tissues. One of the earliest EMT processes in vertebrates is observed in body plan formation, as epiblasts are involuted through the primitive streak to form primary mesenchyme. (C) In response to inflammatory signals, type II EMT is responsible for coordinating wound healing and the deposition of extracellular matrix. (D) Type III EMT is characterized in cancer invasion and metastasis as cells leave the primary tumor to reach secondary sites. Cells with partial EMT characteristics are the most invasive. Created with Biorender.com.

Epithelial-mesenchymal transition is a reversible cellular process in which epithelial cells lose their cell-to-cell adhesion, polarity, and attachment to a basement membrane to become more motile, with mesenchymal features that include increased invasiveness, cell survival, and extracellular matrix remodeling [Figure 1A]^[9]^.

### EMT types

Depending on the biological context in which it occurs, EMT can be classified into three subtypes: Type I, II, and III [Figure 1B–D]^[18]^. The first type was described extensively in the context of embryonic development, where cells must undergo multiple rounds of EMT and its reverse, mesenchymal-epithelial transition (MET), to shape adult tissues and organs^[19]^. That is, from implantation to organ formation, epithelial cells progressively transition through multiple rounds of EMT, which ultimately have the function of generating multipotent mesenchymal cells that will give rise to differentiated cell types^[19]^. Since many molecular processes observed in cancer, including EMT, are a recapitulation of embryonic processes^[20]^, we review developmental EMTs for insights that may prove instructional in ovarian and other cancers. In embryonic patterning, Snail was the first in its family to be identified in Drosophila melanogaster^[21]^.

Alternatively, in vertebrates, the fibroblast growth factor (FGF)-induced expression of Snail was shown to contribute to mesoderm cell fate and somite formation [Figure 1B]^[22–24]^. Specifically, at the paraxial and posterior embryonic mesoderm of mice, Snail represses cadherin 1 (*CDH1*/E-cadherin) during primitive streak formation^[22]^. In turn, downregulation of E-cadherin increases β-catenin availability, thus preparing for Wnt signaling and EMT^[22]^. After delamination from the primitive streak, the paraxial mesoderm mesenchymal cells migrate to the posterior presomitic mesoderm, where they once again encounter high levels of FGF^[25]^. At the determination front, Wnt and FGF signaling decrease and epithelization of somites occurs^[26]^. In response, Snail expression oscillates, thus, participating in the integration of the signaling wavefront with the process of somite segmentation^[23]^, with a key role in secondary body formation (“trunk-to-tail transition” in mice)^[27,28]^. Indeed, several of these embryonic pathways have been validated to play important roles in ovarian cancer. For example, there is evidence that FGF signaling increases aggressiveness and contributes to chemotherapy resistance^[29,30]^.

Due to its inhibitory functions, Snail is classified as a zinc-finger transcriptional repressor^[31,32]^. Its structure is composed of an N-terminal SNAI1/GFI (SNAG) domain, a serine-rich domain, a nuclear export signal, and four C-terminal C2H2 zinc-finger domains^[33]^. In vertebrates, gene repression is achieved through the C2H2 zinc-finger domains that can bind to Ephrussi boxes (E-boxes; CANNTG) found in gene promoter regions^[33]^.

Besides embryogenesis, EMT (type II) has also been observed in the re-epithelialization and extracellular matrix deposition that occur during tissue repair and fibrosis, mainly as a response to inflammation, often mediated by cytokines [Figure 1C]^[34]^. For instance, as a response to inflammatory cytokines released after injury, keratinocytes at the margin of the wound lose their adhesion to each other through the expression of EMT-associated genes, such as fibroblast-specific protein 1 (*FSP1* or *S100A4*), C-X-C motif chemokine ligand 1 (*CXCL1*), actin alpha 2, smooth muscle (*ACTA2*), thrombospondin 1 (*THBS1*), TIMP metallopeptidase inhibitor 1 (*TIMP1*), tropomyosin 1/2 (*TPM1*/*2*), interleukin 6 (*IL6*), brain abundant membrane attached signal protein 1 (*BASP1*), and vimentin (*VIM*); these same factors give them the capacity to migrate from the edge of the wound to fill the area that has been damaged^[35,36]^. Compared to EMT type I, type II usually occurs in adult or maturing tissues, producing mostly fibroblasts instead of mesenchymal cells^[18]^. Snail and its family member *SNAI2* (Slug) are upregulated as a result of growth factor signaling to promote increased cellular proliferation, motility, and matrix remodeling^[34]^. In fibrosis, Qi *et al*. and others recently established that Snail induced partial EMT in a mouse renal fibrosis model and, together with p53-p21-mediated cell cycle arrest, formed a reciprocal loop via the NF-κB pathway, contributing to the advancement of the disease^[37]^. In this way, although EMT is essential for proper developmental biology as well as wound healing, dysfunction of this process, such as prolonged activation, can result in disease processes, such as fibrosis and cancer metastasis.

In cancer specifically, type III EMT is described [Figure 1D]^[18]^. In fact, EMT has been the central hypothesis as to how epithelial primary tumor cells are able to shear off, disseminate, and metastasize to secondary locations^[38]^. Through the expression of master EMT transcription regulators, such as Snail, Slug, Twist family basic Helix-Loop-Helix (bHLH) transcription factor 1 (*TWIST1*), and Zinc finger E-box binding homeobox 1 and 2 (*ZEB1/2*), epithelial cancer cells not only decrease cell-cell adhesion through the inhibition of epithelial markers, like E-cadherin^[31,32]^, they also increase expression of matrix metalloproteinases (MMPs)^[39]^, increase chemotherapy resistance and cell survival^[13]^, and contribute to the production of cancer stem-like cells (CSCs)^[40]^. Thus, these cells have enhanced invasive and stemness properties^[41]^. When comparing EMT type III to types I and II, cancer cells demonstrate phenotypic similarities and are capable of replicating functionalities seen in both subtypes. Namely, they may invoke expression of pluripotency (type I) and mesenchymal markers (type II) and induce tissue invasiveness (type I)^[18]^. While there are context-dependent and mechanistic differences in developmental, inflammatory-mediated, and cancer-associated EMTs, lessons learned in all EMT types have provided valuable insights into the causes of cancer aggressiveness.

It is important to note that, although Snail’s function has been characterized within the different contexts/types of EMT in this review, Slug, Twist, and Zeb1/2, which are the remaining core EMT-transcription factors (EMT-TF), have also been described to broadly induce the EMT program. Aside from their inherent differences in overall structure, regulation, expression patterns, and binding affinities, each EMT-TF also has its own non-redundant functions that are tissue- and context-specific (extensively reviewed in^[33]^).

### EMT spectrum

In the past, EMT was thought to be a binary process; that is, it was thought that epithelial cells would be induced to completely change into mesenchymal cells^[42]^. However, with further studies, intermediate or hybrid states that combine the two phenotypes have been observed, including in ovarian cancer^[43–47]^. These transition states, which are characterized by differential expression patterns of surface markers as well as transcription factors, add to the complexity that is observed within a heterogeneous tumor. In other words, ovarian cancer cells undergoing EMT are more accurately described as cells transitioning through a spectrum that is governed by the factors present within the tumor and its microenvironment^[48,49]^. Furthermore, as observed in pancreatic cancer, in order for metastasis to occur successfully, invasive mesenchymal-like cells are required to return to a more epithelial state for the efficient colonization of secondary tissues^[50]^. Therefore, hybrid EMT states are not only existent but also stable. In fact, Jolly *et al*. expanded on the work done by Watanabe *et al*.^[51]^ and Hong *et al*.^[52]^ on finding transcription regulators, besides the OVO-like transcriptional repressor (*OVOL1/2/3*) family, responsible for the stabilization of hybrid EMT phenotype on a single-cell level^[53]^. Through mathematical modeling and *in vitro* confirmation in lung adenocarcinoma cell lines, they identified grainyhead like transcription factor 2 (*GRHL2*) and microRNA 145 as additional factors responsible for the inhibition of complete EMT^[53]^. Furthermore, Bocci *et al*. detailed the regulatory effect of Numb in preventing full EMT via the NOTCH signaling pathway^[54]^. Their studies also correlated high Numb expression with poor survival in ovarian cancer^[54]^. More specifically to HGSOC, Varankar *et al*. established the functional relevance of the Tcf21-Slug axis in promoting cellular plasticity in EMT^[55]^. Such models that establish the stability of the hybrid state are still evolving within ovarian cancer literature. By combining genetic and biophysical parameters, these models would allow for a more quantitative evaluation of partial EMT dynamics, which could then be tested experimentally.

Another study by Ocana *et al*. demonstrated the importance of reversibility of EMT with their paired related homeobox 1 *(PRRX1)* overexpression experiments in zebrafish and chicken embryos as well as breast cancer cells^[56]^. Prrx1 is an EMT inducer that works independently from conventional EMT transcription factors. When it is overexpressed, full EMT can be achieved; however, if the overexpression is maintained, cells are unable to metastasize due to the loss of cancer cell stemness, which is mostly responsible for the tumor-initiating properties at distant sites^[56]^. In this way, through a highly complex sequence of changes in marker expression and presentation, each EMT state within the spectrum has its own level of proliferation, invasion, plasticity, stemness, metastasis, and resistance to therapy^[43]^. By understanding the different EMT states, more insight could be gained in the search for potential therapeutic agents that would be patient-centered and effective in eradicating the disease.

### Ovarian cancer subtype classifications

To better represent the complex and diverse phenotypic variety of ovarian cancer cells observed in patients, there have been attempts to classify commonly used cell lines into an EMT spectrum^[47,57]^. For instance, Huang *et al*. categorized cell lines based on their immunofluorescence expression patterns of E-cadherin, pan-cytokeratin, and vimentin^[47]^. Based on these markers, cells were divided into four groups: epithelial, intermediate epithelial, intermediate mesenchymal, and mesenchymal^[47]^. More specifically, the expression of E-cadherin determined the major grouping of epithelial versus mesenchymal, then the positive expression of vimentin or pan-cytokeratin led to the further classification of intermediate epithelial and mesenchymal states, respectively^[47]^. Similarly, Strauss *et al*. used E-cadherin, Tie2, prominin 1 (CD133) and CD44 to place cells into E, E/M, and M subgroups^[46]^. Further functional analyses, with viability and spheroid assays, determined that each EMT group had different characteristics *in vitro*, emphasizing the importance of distinguishing cells along the EMT spectrum^[47]^.

With the increasing emphasis on translatability and the advancement of genomics, other groups focused on directly analyzing patient tumor samples to better characterize the different molecular subtypes observed in ovarian cancer. Whole genome and whole exome sequencing have been used to identify genomic alterations in ovarian cancer, including mutations, copy number variations, and structural variants^[58]^. These have yielded key insights regarding genetics, tumor heterogeneity, and chemoresistance. RNA sequencing has facilitated gaining new knowledge of several characteristics of ovarian cancer, and its integration with proteomic, metabolic, and histopathological data has allowed the development of prediction algorithms for grade, transcriptomic subtype, and chemotherapy response^[59,60]^. Using differential gene expression clustering, the pioneering work of Tothill *et al*. classified 285 patient samples into six molecular subtypes, C1-C6^[61]^. Of these six subtypes, the majority of high-grade serous tumors were segregated into subtypes C1 (high stromal response), C2 (high immune response), C4 (low stromal response), and C5 (mesenchymal development)^[61]^. Not only did these subtypes represent the molecular heterogeneity among patients, but they also provided further insight into the different histopathological and patient survival characteristics. Later, in 2011, the Cancer Genome Atlas Research Network published its integrated genomic analyses of 489 HGSOC patient samples^[62]^. Similar to the results obtained by Tothill *et al*., through consensus clustering, this study also identified four different subtype clusters for HGSOC, which were classified as differentiated, immunoreactive, mesenchymal, and proliferative^[62]^. Most recently, Tan *et al*. performed their own meta-analysis on over 1,500 samples of EOC, which were also divided into five subtypes that can be identified as Epithelial-A, Epithelial-B, Mesenchymal, Stem-A, and Stem-B^[44]^.

Although it seems that each group provided its own distinct stratification of patient samples, Tan *et al*. also demonstrated the comparability of these classifications^[44]^. Even though intrinsic biological properties may sometimes lead to a discrepancy in subtype prediction, it is possible to make a general integration of the knowledge obtained through these different studies to provide a greater understanding of the different molecular subtypes [Table 1]. For instance, the Epithelial-A subtype corresponds to the C3 and differentiated subtypes, the Epithelial-B to the C4 and C2, as well as the differentiated and immunoreactive subtypes, the Mesenchymal to the C1 and mesenchymal subtypes, the Stem-A to the C5 and the proliferative subtypes, and Stem-B to the C6 subtype^[44]^. Further confirmation of subtype classification overlap (the Cancer Genome Atlas and Tothill) was described in the genomic and transcriptomic characterization of HGSOC performed by Hollis *et al*^[63]^. Their study also revealed that the immunoreactive/C2 subtype had enrichment for BRCA1/2 DNA repair associated (*BRCA1/2*) mutations and the proliferative/C5 subtype had high rates of CCNE gain^[63]^. With all these different classifications, the recurring theme is that HGSOC is indeed complex and heterogeneous, and higher Snail expression can be narrowed down to two main subtypes, Mesenchymal and Stem-B, of which Mesenchymal is the most applicable to serous invasive tumors^[64]^.

A limitation of the genomic studies mentioned above is that their expression patterns have been mostly observed in whole tumor samples; therefore, a single-cell approach would provide a more thorough perspective, especially in terms of individual cell gene expression within the different subtypes of EMT. Currently, there are very few studies characterizing EMT at the single-cell level in HGSOC, with some of the most recent ones being on growth factor-induced EMT with the cell line OVCA420 and EOC ascites tumor cell clusters^[64,65]^. Insights from Cook *et al*. highlight the context specificity of EMT-related gene expression patterns^[64]^. Their time course experiments demonstrated that EMT is not a linear process but a multistep process with discrete transcriptional events, further confirming the concept of an EMT spectrum. Of interest, Snail and other canonical EMT markers and transcription factors had differential expression in only a small number of time course conditions, meaning that their expression patterns showed inconsistent involvement in the transition^[64]^. Within ascites samples, Kan *et al*. used a panel of 53 EMT genes to classify single cells into three subtypes: EPCAM+ (epithelial cell adhesion molecule^[65]^; marker for epithelial tumor cells), CD45+ (PTPRC, protein tyrosine phosphatase receptor type C; marker for leukocytes), and EPCAM-/CD45− (marker for CAFs). Their results suggested that ascites clusters that contained a mixture of tumor cells and cancer-associated fibroblasts had higher proliferation capacity and anoikis protection. The epithelial cancer cells within these heterogenous ascites clusters are also enriched in EMT hallmark genes^[65]^. In patient samples, single-cell RNA sequencing revealed HGSOC heterogeneity, and an EMT-associated signature allowed prediction of patient outcomes^[66]^. Given these new findings, further confirmation is required in additional cell lines and patient-derived samples, as well as other genomic approaches.

A concern that has arisen recently in colorectal and other cancers is the possibility that the EMT signature observed in tumors reflects its stromal composition, rather than genes expressed in tumor cells^[67,68]^. As shown in work from the Wong and Sood labs in 2021/2022, the use of SpatioImageOmics and spatial transcriptomics can aid in clarifying this uncertainty^[69,70]^. Combining imaging mass spectrometry and location-specific transcriptomics revealed the spatial relationships between tumor, immune, and stromal cells in advanced HGSOC^[70]^. In HGSOC, while stromal cells, such as myofibroblasts and mesenchymal cells, have been mapped with EMT-like cells to similar clusters (poor response to neoadjuvant chemotherapy) through spatial transcriptomics, cancer-associated fibroblasts have not, indicating that their transcriptional signatures are different^[69]^. The approaches utilized in such studies of the tumor microenvironment and cancer cell interaction transcriptomics are still in their early stages and have the limitation that they have yet to indicate definitive directional/causative progression through the EMT program. These important recent technological advances have made a large contribution towards clarifying some of the many ill-defined aspects of HGSOC biology. In fact, these studies emphasize the complexity of EMT dynamics and the inadequacy of relying on “hallmark” EMT scores based on expression patterns observed from earlier studies. In order to individualize and improve therapeutic strategies, a greater effort is needed to integrate knowledge obtained from different -omics perspectives with the basic molecular findings that characterize important mechanisms relevant to the progression of the EMT program.

Since its initial observation in early development, EMT has been further characterized in different contexts, one of which is cancer progression. In ovarian cancer, heterogeneity is often observed. Although characterization efforts have uncovered intricate complexities, EMT subtype classification has exhibited a correlation with different degrees of cancer aggressiveness. Snail, one of the master regulators of EMT, was shown to be highly expressed in specific subtypes of ovarian cancer. In the past years, there have been several efforts to establish a mechanistic pathway that delineates the downstream functions of Snail. Within epithelial ovarian cancer, Imai *et al*. were one of the first to observe a negative correlation between Snail and E-cadherin expression under hypoxic conditions^[71]^. Since then, many other groups have further explored Snail’s role in increasing ovarian cancer aggressiveness - namely its effect on pathways related to cancer migration and invasion, stemness, and chemoresistance. In the following sections, we aim to summarize the most recent findings within this field that will potentially impact the future of novel therapeutics development.

## CANCER AGGRESSIVENESS

Given that EMT morphologically and cell biologically equips cancer cells for migration and invasion, it is expected to play a role in aiding mobility as a carcinoma cell leaves the primary tumor site, and its invasion through the basement membrane and subsequent metastasis to secondary sites. In fact, cell invasion is a culmination of many signaling pathways, such as transforming growth factor beta (TGF-β), fibroblast growth factor, bone morphogenetic protein, activin, parathyroid hormone-related peptide, GLI, Notch and Wnt signaling pathways, that contribute to and overlap with EMT. Specifically, HIF1, Src, Ras, and Ets1 transcription factors have been shown to activate Snail signaling via interactions with the MEK/ERK, PI3K, and SMAD signaling pathways^[72–74]^. Furthermore, it is important to note that while EMT is an important contributor to cancer invasion, the tumor microenvironment also plays a significant role in EOC progression. In fact, there are many factors at play, including extracellular matrix components, adipocytes, endothelial, mesothelial and mesenchymal stem cells, and immune response cells^[75–77]^. Their normal physiological roles are either thwarted or used for the benefit of the tumor.

### Cancer invasion and metastasis

For ovarian cancer cells to become invasive, they have to perform matrix remodeling to access their secondary location^[78]^. In this scenario, stromal cells, such as mesothelial cells, cancer-associated fibroblasts (CAFs), and mesenchymal stem cells, are known contributors to the regulation of extracellular matrix (ECM) composition^[76,79]^. Moreover, a layer of mesothelial cells is typically the first defensive barrier that ovarian cancer cells must cross to invade the basement membrane, which is composed of a variety of extracellular matrix structural proteins^[77]^. While it is the interaction between cancer cells and stromal cells that regulates the metastatic process, in addition to the proteinases produced by CAFs, cancer cells themselves are capable of producing the digestive enzymes that degrade the ECM barriers and reshape their tumor microenvironment^[80]^.

Within the ECM, two major groups of proteins are known to determine the extent of invasion and metastasis: extracellular matrix structural proteins, like collagen, laminin, and fibronectin, and cell surface receptors and ligands^[81]^. Since matrix metalloproteinases (MMPs) are known to degrade the first group of proteins, extensive focus has been given to them in the field of cancer metastasis^[81,82]^. For instance, high MMP19 and MMP20 expression could serve as predictors of poor prognosis in ovarian cancer since they have been shown to increase invasiveness^[81]^. Likewise, MMP2, MMP9, MMP14, and TIMP metallopeptidase inhibitor 2 (*TIMP2*) have also shown similar results, thus indicating the relevance of matrix remodelers in cancer invasion^[81]^. In addition to their role downstream of EMT, MMPs have been shown to positively reinforce the induction of EMT in epithelial cancer cells, thus, enhancing the invasive and metastatic properties of the primary tumor^[83–85]^.

In the early stages of EMT, Snail is known to inhibit the expression of E-cadherin (*CDH1*), tight junction protein 1 (*TJP1* or *ZO-1*), and occludin (*OCLN*) *in vitro*^[86]^; however, its further mediation of ovarian cancer invasion and migration is complex, involving the regulation and modification of a variety of intracellular and extracellular factors [Table 2]. Within cervical cancer cell lines, Snail function has been associated with the differential expression of many genes *in vitro*. Jin *et al*.^[87]^ demonstrated that Snail knockdown increased the expression of protease inhibitors and cell adhesion molecules [e.g., apoptosis inhibitor 5 (*API5*); *TIMP3*; erythrocyte membrane protein band 4.1 like 4.B (*EPB41L4B*); secreted phosphoprotein 1 (*SSP1*); fos proto-oncogene, AP-1 transcription factor subunit (*FOS*); integrin subunit alpha 6 (*ITGA6*); metastasis associated 1 *(MTA1*); caspase 8 (*CASP8* or *FLICE*); and Cadherin 1 (*CDH1*)], and decreased the expression of genes related to invasion and migration [serpin family B member 5 (*SERPINB5*); neural cell adhesion molecule 1 (*NCAM1*); *MMP2*; elastase, neutrophil expressed (*ELANE*); *MMP7*; nerve growth factor (*NGF*); *S100A4*; and *MMP1*]^[87]^. Given this evidence, future genomic studies in the form of whole-genome chromatin immunoprecipitation sequencing (ChIP-seq) for Snail binding sites in ovarian cancer cells derived from patient samples would be highly beneficial in determining the direct and indirect regulatory mechanisms of Snail. Such molecular data would assist in the identification of possible Snail inhibition sites.

**Figure 2.**
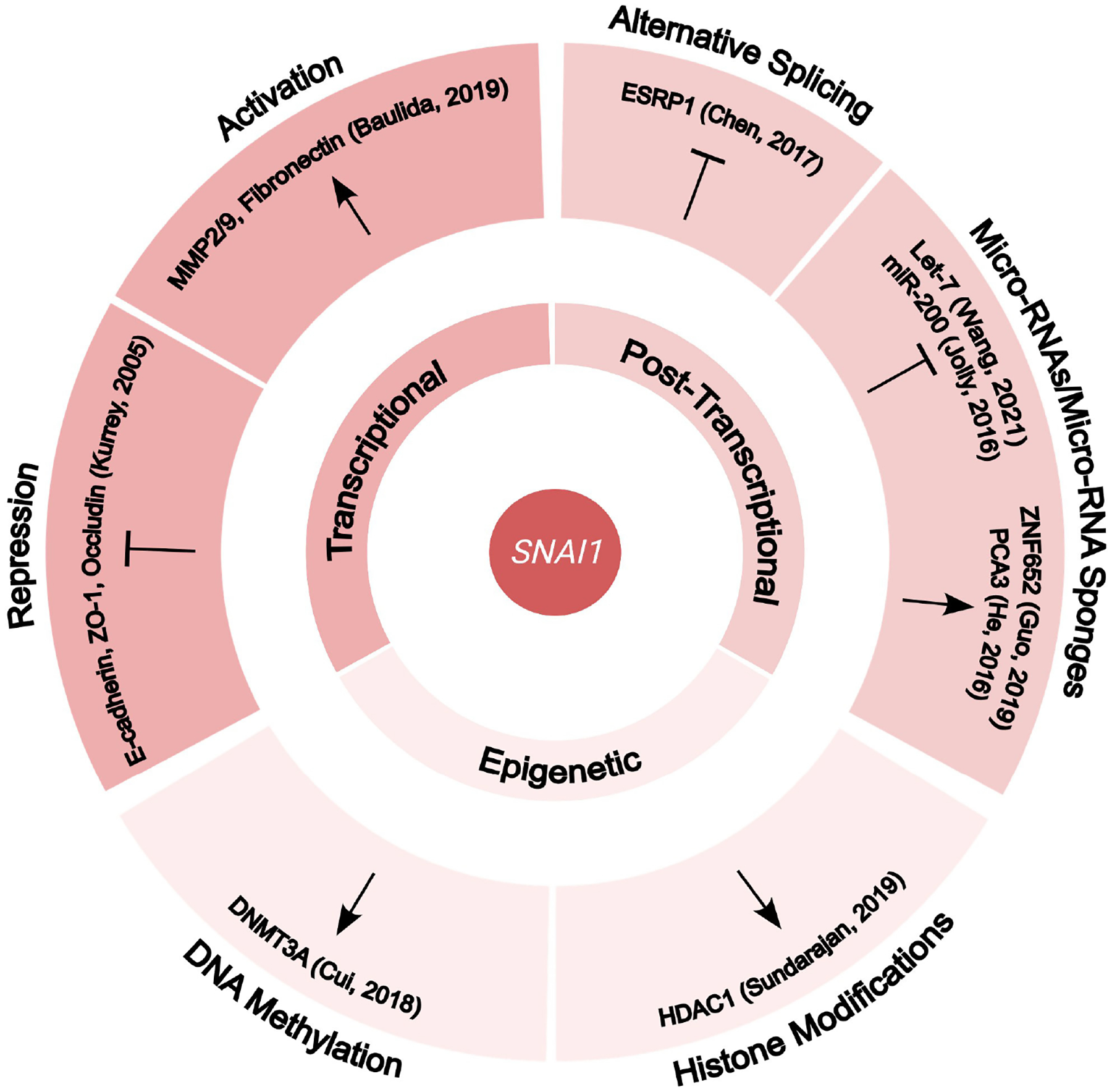
Downstream functions of Snail in different types of carcinomas. Snail has been shown to function at multiple levels - transcriptional, post-transcriptional, and epigenetic (specific examples shown). In addition to its function as a transcriptional repressor of epithelial markers in EMT, Snail can activate the transcription of matrix metalloproteinases and mesenchymal factors. At the post-transcriptional level, Snail also affects the alternative splicing of CD44 by inhibiting ESRP1 and the expression of micro-RNAs and micro-RNA sponges. Lastly, in terms of epigenetic modifications, Snail alters histone modifications and DNA methylation by its interaction with histone deacetylases (HDACs) and DNA methyltransferases (DNMT).

Besides its immediate role in transcriptional regulation^[86,108]^, Snail can indirectly control cancer invasion by binding to factors involved in post-transcriptional modifications, such as splicing factors^[109]^ and non-coding-RNAs^[12]^, as well as factors responsible for epigenetic modifications^[103,110]^ [Figure 2]. For example, Snail can bind to the promoter of the epithelial splicing regulatory protein 1 (*ESRP1*), repressing its function in a cervical cell line. In turn, downregulation of *ESRP1* can lead to the alternative splicing of CD44 molecule (*CD44*), from *CD44v* to *CD44s*, increasing *in vitro* and *in vivo* invasiveness^[109]^. Additionally, Snail has also been shown to repress the function of *MIRLET7* (let-7)^[12]^ and *MIR34* (miR-34)^[111]^ family members *in vivo* and *in vitro*, respectively, ultimately leading to an increase in cancer cell stemness, invasiveness, and metastasis. Lastly, Sundararajan *et al*. demonstrated Snail’s ability to recruit histone deacetylase (HDAC) co-repressors to inhibit Slug *in vitro*, demonstrating how EMT transcription factors can regulate each other to perform their context-dependent functions^[103]^.

It is important to note that although cancer invasion can be thought of as a single-cell event - in the sense that cells individually exit the primary tumor through EMT - more recent studies have shown growing evidence for collective cell invasion in ovarian cancer^[65,79,112]^, in which a group of cells can collectively migrate away from the primary tumor to a secondary tissue. Further, in many malignancies, such as breast cancer, colorectal cancer, and EOC, the hybrid EMT state has been found to enhance metastasis via collective, clustered cell migration [Figure 1D]^[113–115]^. The ability of these cells to invade local tissues, modify the surrounding ECM, as well as adapt and shape the tumor microenvironment (TME) results in worse clinical outcomes and poor patient prognosis^[116–118]^. In squamous cell carcinoma, Li *et al*. observed collective migration as a result of Snail activating the expression of claudin 11 (*CLDN11*)^[119]^. In addition to the more broadly accepted route of metastasis to the peritoneal cavity, ovarian cancer may spread hematogenously^[120]^, and patients with circulating tumor cells in whole blood have shown worse clinical prognosis^[121]^. Nevertheless, further studies are still needed to determine whether Snail expression affects collective invasion in ovarian cancer metastasis.

In summary, Snail has been shown to induce cancer invasion and migration in various cancer types, including ovarian cancer. Its downstream functions range from transcriptional and post-transcriptional to epigenetic regulations. Although targeting this transcription factor has proven challenging as expected, strategies to inhibit its protein-protein interactions are promising, demonstrating its potential in decreasing ovarian cancer aggressiveness.

### Cancer stemness

Stemness refers to the acquisition of stem cell characteristics, which include the capacity for self-renewal as well as the ability to differentiate for the preservation of balance between quiescence and proliferation^[122]^. Evidence for parallels between normal stem cell biology and cancer biology was first obtained in 1994, when a study on human acute myeloid leukemia revealed the presence of leukemia-initiating cells within the whole cell population^[123]^. More than ten years later, cancer stem cells (CSCs) were identified in epithelial ovarian cancer^[124]^. These CSCs exist as a small subpopulation in malignant ovarian tumors and are generally thought to be an important contributor to cancer recurrence due to their ability to confer chemotherapy resistance and clonal growth leading to metastasis formation^[125]^. For these reasons, ovarian cancer stem cells have become an attractive target for the development of novel therapies designed for complete eradication of the disease. We focus here on mechanisms by which Snail contributes to stemness, an area of ongoing research.

**Figure 3.**
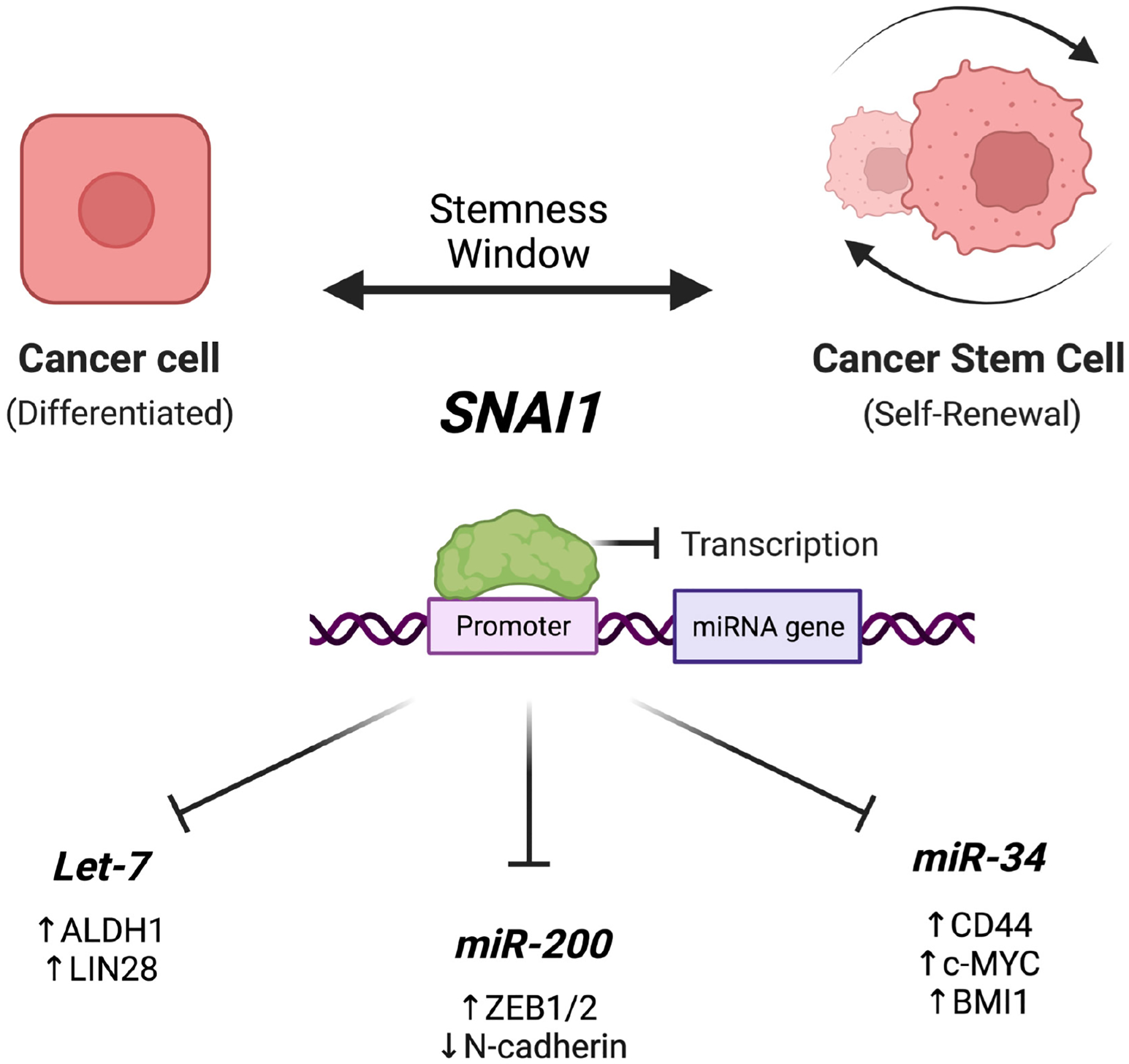
Development of cancer stemness through downstream effects of SNAI1 on miRNAs. During the transition from a differentiated cancer cell into a cancer stem cell, there is a transitional period termed the “stemness window” in which several molecular events occur that drive a stem cell-like phenotype. Snail, an EMT master regulator, acts to inhibit the transcription of several regulatory microRNAs. These include the let-7 family, regulators of differentiation, the miR-200 family, regulators of EMT, and the miR-34 family, regulators of apoptosis. Through the inhibition of these microRNAs and the dysregulation of their targets, several genotypic and phenotypic changes are induced (examples noted below the miRNAs), thus driving the development of stemness traits. These include the ability to self-renew, differentiate, and initiate tumor formation. Created with Biorender.com.

Some downstream targets of Snail contributing to stemness have been identified and their function demonstrated. Several microRNAs with known roles in stemness have been found to be regulated by Snail; we will focus on the *MIRLET7*, *MIR34*, and *MIR200* families (miR-200), miRNAs that are associated with tumor suppression^[12,53,95,96]^ [Figure 3]. Siemens *et al*. reported that, in a lung cancer cell line, *MIR34A/B/C* is transcriptionally repressed by Snail, and in a double negative feedback loop, *MIR34A/B/C* inhibits Snail^[95]^. *MIR34A* expression resulted in downregulation of the stemness factors BMI1 proto-oncogene polycomb ring finger (*BMI1*), CD44, CD133, olfactomedin 4 (*OLFM4*), and MYC proto-oncogene bHLH transcription factor (*MYC*) in a colon cancer cell line^[95]^, clearly connecting this axis to stemness. Further confirming this finding, in an ovarian cancer study, it was found that MIR34A acts as a tumor suppressor by targeting proteins, such as Snail, involved in apoptosis, proliferation, metastasis, and stemness^[111]^. Snail also induces, and *MIR34A* inhibits, zinc finger protein 281 (*ZHF281)*, a protein demonstrated to regulate and maintain pluripotency by interacting with transcription factors associated with stemness [*NANOG*, POU class 5 homeobox 1 (*POU5F1/OCT4*), SRY-box transcription factor 2 (*SOX2*)]^[126]^.

Another tumor suppressor miRNA family is the miR-200 family. While many studies have established the double-negative feedback loop that exists between the EMT transcription factors, *ZEB1/2*, and the miR-200 family in the context of EMT^[97,98,127,128]^, Snail expression has also been linked to the regulation of miR-200 family members^[96,98]^. In a colorectal cancer cell line, Snail was shown to directly bind *miR200c*’s promoter region and thus modulate its expression^[98]^. Diaz-Lopez *et al*. not only confirmed these results with *miR200f*, but also established that Snail can regulate CpG methylation in *miR200f* loci through the use of MDCK cells in a typical EMT modeling system^[96]^. The mir200 family contributes to stemness by increasing clonogenicity and Wnt signaling, and thus increases tumor initiation capacity^[129]^.

Focusing on epithelial ovarian cancer, Wang *et al*. found that Snail contributes to stemness by directly inhibiting several *let-7* family members^[12]^. Induction of Snail expression via epidermal growth factor (*EGF*) and Snail overexpression via viral transduction resulted in a decrease in the expression of four *let-7* family members, an increase in the expression of several stemness markers [*LIN28A*, Nanog homeobox (*NANOG*)*, OCT4* (*POU5F1*), high mobility group AT-hook 2 (*HMGA2*)], and an increase in self-renewal and growth as evidenced by spheroid assays. Snail knockdown, subsequently, had the opposite effects in these cell lines as well as in patient-derived high-grade serous ovarian cancer cells both *in vitro* and *in vivo*. Further, ChIP analysis displayed that Snail directly binds to *let-7* promoters^[12]^. These findings (validated by luciferase assays) reveal that Snail directly represses *let-7* transcription and subsequently promotes the acquisition of stem cell-like properties in cancer. This miRNA plays an important role in ovarian biology^[130]^, and it remains to be seen whether the Snail/*let-7* axis is also active in development and in reproductive organs. Another EMT transcription factor, *Twist1*, also inhibits *let-7* transcription^[131]^; thus, the connection between EMT and stemness via *let-7* inhibition is strong.

The action of Snail on *let-7* may contribute to destabilization of the differentiated state. Snail inhibition of *let-7*^[12,92]^ has been found to allow the upregulation of *let-7*’s pluripotency and oncogene targets such as *LIN28*, *RAS*, *MYC*, and many others. *Let-7* inhibition is required for reprogramming somatic cells to pluripotency^[132]^. Since *let-7* maintains differentiation, its inhibition may similarly be necessary for reprogramming-like events that introduce stemness in cancer cells.

For many years, epithelial-mesenchymal transition was not only thought to be largely responsible for the development of stemness traits^[40,133]^, but several studies also showed the reverse: stemness promoting EMT^[134,135]^. With the increasing knowledge on cancer plasticity, however, the link between EMT and cancer stemness has become much more complicated. That is, depending on the levels of EMT transcription factors being expressed, the location of the “stemness window” can be flexible within the EMT spectrum^[136]^. In specific, Jolly *et al*. found that miR-200’s inhibition of lin-28 homologs (*LIN28A/B*) can link EMT/MET with stemness via the double-negative feedback loops existing between miR-200/zinc finger E-box binding homeoboxes (*ZEB1/ZEB2)* and *LIN28*/*let-7*^[137]^. In such a context, Snail appears to be a key player in the relationship between EMT and CSCs; many publications have established this role of Snail (reviewed in^[138]^).

One way by which Snail may mediate stemness involves its role in partial EMT (also called the hybrid epithelial/mesenchymal state), in which cells express both epithelial and mesenchymal characteristics and are more invasive^[41]^. This concept has been widely established, as cells that exist in a purely epithelial or mesenchymal state do not exhibit stemness traits^[43]^. Furthermore, cells undergoing collective migration (with characteristics of partial EMT^[139]^) have characteristics of stem cells, and their presence correlates with cancer progression^[119]^. Snail has also been found to stabilize this state within breast cancer cells, suggesting that partial EMT is an aspect of cancer stemness^[140]^. Surprisingly, Snail expression is lower in both breast and ovarian cancer cells that are on the mesenchymal end of the spectrum, indicating that it plays an important role in engaging collective cell migration in the hybrid state^[140]^. Cells undergoing collective migration are largely found in the partial EMT state within a TME structured by Snail-expressing CAFs, bringing together Snail expression, stemness, plasticity, and metastasis and demonstrating the multifaceted aggressiveness mediated by this EMT transcription factor^[139]^.

Collectively, these studies, and others, elucidate the significance of Snail in promoting stemness within the CSC population. In EOC, research has revealed that Snail is important for enhancing invasion, migration, and survival via loss of epithelial markers, gain of mesenchymal characteristics, and modulation of the cytoskeleton. As evidenced by the multiple studies completed in other cancer types, further work must be performed in the context of EOC to determine specific mechanisms of Snail-induced EMT and stemness. Snail’s direct and indirect regulation of developmental and differentiation regulators, such as *let-*7, miR-34, and miR-200, are just some of the crucial mechanisms for exerting stemness in cancer. It is reasonable to suggest that Snail’s role in EMT, and subsequent role in stemness, is one major factor in the development of metastasis, recurrence, and drug resistance^[12,13,141]^.

### Metabolic effects

Another way that Snail results in increased aggressiveness is by contributing to the metabolic reprogramming of cancer cells (reviewed in^[142]^). In breast cancer, Snail directly regulates components of pathways important for the metabolic changes associated with the glycolytic switch associated with CSC^[100,143]^ [Table 1]. Many of the proteins upregulated by EMT in an HGSOC cell line, analyzed by proteomics and bioinformatics, play roles in metabolism^[144]^. With the induction of EMT with EGF, Grassi *et al*. identified a list of 30 proteins that were associated with the biological process of metabolism^[144]^. Within this set of proteins, asparagine synthetase (*ASNS*) and signal transducer and activator of transcription 3 (*STAT3*) have been associated with asparagine synthesis in chemoresistant cells and aerobic glycolysis within stem cells^[142]^, respectively. This is consistent with results from the Ahmed lab showing that in chemoresistant cells in which an EMT phenotype is observed, the metabolic profile is altered, verified by functional assays, and validated in patient samples^[145]^. Studies on the role of EMT in the metabolic control of CSC in HGSOC are lacking, but metabolic pathways in ovarian CSC are known to differ from the non-CSC pool^[146]^, although whether these pathways are downstream of EMT is not yet known. Thus, Snail is expected to control genes with roles in metabolism in HGSOC, and evidence suggests this in CSC specifically.

### Chemotherapy resistance

The cornerstone treatments in front-line therapy for EOC are platinum- and taxane-based regimens^[147]^. Although these regimens are effective in most patients in first-line settings in inducing partial or complete remission, patients often develop chemoresistance^[8]^. Poly (ADP-ribose) polymerase inhibitors (PARPi) have become commonly utilized, mainly in the maintenance setting but also as monotherapy in the recurrent setting after multiple lines of chemotherapy^[148]^. Acquired resistance to PARP inhibitors does occur, with multiple cellular pathways implicated^[149]^.

Multiple mechanisms have been proposed as to why and how chemoresistance occurs, but in this section, we focus on the role of EMT. One of the methods by which EMT contributes to chemoresistance is through the upregulation of multi-drug resistance transporters that can remove drugs from the cell^[150,151]^. In fact, Saxena *et al*. performed a thorough study of the relationship between EMT and ATP-binding cassette (ABC) transporters in breast cancer^[151]^. They observed that Snail, Twist, and forkhead box C2 (*FOXC2*) were able to repress the promoter activity of *ABCC5* in luciferase assays^[151]^. Furthermore, chromatin immunoprecipitation revealed that Twist bound to E-boxes in *ABCC4* and *ABCC5*^[151]^. In a similar manner, Wang *et al*. found that Snail directly regulates *ABCB1* transcription in colorectal cancer^[150]^.

In ovarian cancer, EMT has also been linked to chemotherapy resistance^[13,114,152,153]^. Haslehurst *et al*.^[152]^ showed that *in vitro* knockdown (KD) of Snail and Slug in cisplatin-sensitive and -resistant cell line pairs, A2780 and A2780-cis, respectively, resulted in a restoration of sensitivity in the resistant cell line^[152]^. These results were confirmed by Hojo *et al*. in the HGSOC cell line, OVCAR8; Snail KD cells were then injected into the ovarian bursae of Nude mice after Snail KD, resulting in a significant decrease in tumor burden^[141]^. In an elegant study performed by Sundararajan *et al*., Snail was overexpressed in OVCA420 (epithelial-like ovarian cancer cell line) and OVCA429 (intermediate epithelial-like/hybrid ovarian cancer cell line) to mimic EMT subtype progression in ovarian cancer^[154]^. By simulating the sequential changes that must occur in Snail-driven EMT, they were able to observe that Snail overexpression had a different effect depending on the initial subtype classification^[154]^. That is, OVCA420 (epithelial) with Snail overexpression had higher proliferation, but lower resistance to anoikis, while OVCA429 (hybrid) with Snail overexpression had lower proliferation and invasion, but higher resistance to anoikis^[154]^. In other words, progression through Snail-driven EMT eventually leads to greater cell-death resistance. This study has larger implications for chemoresistance because the initial EMT subtype classification of a patient’s tumor could potentially affect therapy response.

Furthermore, to better determine the mechanism by which Snail affects chemoresistance, Kurrey *et al*. performed whole-genome analyses of ovarian cancer cells that revealed Snail’s potential downstream targets^[13]^. From their study, it was determined that Snail can mediate resistance in two ways: through the repression of genes involved in the p53-mediated apoptosis pathway [ATM serine/threonine kinase (*ATM*), BCL2 binding component 3 (*BBC3*), phosphatase and tensin homolog (*PTEN*)], and/or through the activation of genes related to cancer stemness/pluripotency [*NANOG, CLDN3, OCLN, HDAC1*, transcription factor 4 (*TCF4*)]^[13]^.

In summary, by expressing Snail, cells undergoing EMT acquire resistance to chemotherapeutic agents. Within ovarian cancer, its expression has been associated with anoikis resistance and the regulation of apoptosis and stemness pathways. Together, these studies indicate the importance of understanding the role of Snail in promoting chemotherapy resistance and indicate the urgency of identifying these underlying mechanisms.

## STRATEGIES FOR TARGETING SNAIL

By targeting Snail transcription or translation, its downstream effects on cancer aggressiveness could potentially be reduced. Direct Snail inhibition has been accomplished only via an RNAi approach in which Snail is targeted via mesoporous silica nanoparticle-delivered siRNA. This strategy decreases Snail expression, reducing tumor burden in a patient-derived xenograft model of HGSOC^[12]^. There have been very few attempts to pharmacologically inhibit Snail function. The approaches published so far inhibit the interaction of Snail family transcription factors with protein or DNA binding partners. Namely, cobalt(III)-Ebox conjugate inhibits Snail activity by binding to its Ebox regulatory sequence, decreasing invasiveness in breast cancer^[155]^. Chemicals that disrupt the interaction of Snail and wild-type p53 (GN25 and GN29) were explored for their ability to reactivate the tumor suppressor functions of p53. These inhibitors resulted in reduced *in vivo* metastasis of human lung adenocarcinoma cell line, A549^[156]^, pointing to their potential use in Snail-overexpressing tumors with a wild-type p53 allele. Similarly, Parnate disrupts the interaction of lysine-specific demethylase 1 (*LSD1*) with Snail/Slug’s SNAG domain, resulting in a decrease in migration and invasion of colorectal cancer cells, an effect that resulted in reduced metastasis in an orthotopic breast cancer model^[157,158]^. A molecule that inhibits Snail’s interaction with CBP/p300 (CYD19), which leads to proteasomal degradation of Snail and prevention of wild-type p53 repression, reduces tumor growth and metastasis of colorectal cancer xenografts^[159]^. Likewise, the antibiotic trimethoprim inhibits the Snail/CBP/p300 interaction in colorectal and breast cancer cell lines, reducing viability, preventing EMT, and inhibiting metastatic tumor growth^[160]^. Most recently, dual inhibitors targeting Snail and histone deacetylases have been reported, and resulted in modestly reduced Snail protein levels as well as antiproliferative activity in solid tumor cells including ovarian^[161]^. These few studies highlight not only the potential of Snail inhibition in decreasing cancer invasion and metastasis, but also the lack of successful approaches leading to clinically relevant strategies. Also of note is the necessity of additional alternatives that could target p53-mutated cancer types, such as HGSOC.

## CONCLUDING REMARKS

With increasing evidence, the complexity of EMT is being revealed in cancer progression. We have summarized the role of Snail in development and in cancer, emphasizing its transcriptional, post-transcriptional, and epigenetic functions. Multiple studies have demonstrated that Snail clearly plays a role in determining ovarian cancer aggressiveness, and its role in the hybrid epithelial/mesenchymal state seems to be prominent in stemness and migratory phenotypes. Recent results emphasize that Snail’s actions result in stem cell characteristics, and these mechanisms are starting to be understood: inhibition of tumor suppressor miRNAs are major components. Snail directly regulates factors that contribute to the metabolic changes seen in cancer cells. The expression of Snail also contributes to resistance to chemotherapy. Thus, Snail inhibition is a strategy for the prevention of recurrence in ovarian and other carcinomas. While the superficial outcome of Snail-driven EMT may result in cellular morphological change, its functions have deeper ramifications, as its expression can result in greater cancer invasion, stemness, and chemoresistance. Thus, as new therapeutic approaches are explored, Snail and its mechanistic intricacies should be considered.

## Figures and Tables

**Table 1.** Epithelial ovarian cancer subtype classification and characterization. Subtype classification overlap and clinicopathological correlation adapted from Tan *et al*. (2013)^[44]^ with permission from authors. Gene expression profiles combined from Tan *et al*. (2013)^[44]^, Tothill *et al*. (2008)^[61]^, and The Cancer Genome Atlas (TCGA; 2011)^[62]^. Snail subtype expression levels obtained from Tan *et al*. (2015)^[44]^

Tan *et al*. (2013)^[44]^	Tothill *et al*. (2008)^[61]^	TCGA (2011)^[62]^	HGSOC distribution	Predominant stages	Gene expression profiles		Snail expression
Epithelial-A	C3	Differentiated	7%	I or II	*MAPK* genes (e.g., *SERPIN5A*, MAP3K5)	Epithelial genes (e.g., *CDH1, EPCAM*, KRTs, *CD24*)	Low
Epithelial-B	C4	Differentiated	30%	I or II	Inflammatory and ig genes (e.g., *MHC*)		Low
	C2	Immunoreactive	30%	I or II	Inflammatory and ig genes (e.g., *MHC*)		Low
Mesenchymal	C1	Mesenchymal	32%	III or IV	Fibroblastic/Mesenchymal and inflammatory genes (e.g., *PDGFRA, VCAM1, ZEB1, TWIST1, FN1)*		High
Stem-A	C5	Proliferative	25%	III or IV	Developmental, proliferation, ECM, and sternness genes (e.g., *LGR5, MYCN, NCAM, CDH2, HOXs)*		Low
Stem-B*	C6*		5%	I or II	Sternness genes and β-catenin/LEF/TCF targets (e.g., *PROM1, CD44, MMP7)*		High

* Stem-B/C6: mostly non-serous ovarian cancer.

**Table 2.** List of reported targets of SNAI1 and their function in the cell. All listed targets of SNAI1 were validated with luciferase assay and/or chromatin immunoprecipitation

	Target	Target function/role	Cell type (system)
**Transcriptional Repression**	Claudin 1 (*CLDN1*)	Tight junction component	Epithelial cells^[88]^
	Crumbs cell polarity complex component 3 (*CRB3*)	Epithelial polarity, apical membrane formation, tight junction component	mdck ^[89]^
	CYLD lysine 63 deubiquitinase (*CYLD*)	Tumor suppressor, deubiquitinator	Malignant Melanoma^[90]^
	Cadherin 1 *(CDH1)*	Epithelial state maintenance, adherens junction component	Human carcinoma, epithelial tumor cell lines^[31,32]^
	LLGL scribble cell polarity complex component 2 (*LLGL2*)	Cell polarity	Breast cancer cells, HEK-293T^[91]^
	MicroRNA Let-7 family (*MIRLET7*)	MicroRNA; Differentiation maintenance	Ovarian cancer, fibroblasts^[12,92]^
	Mucin 1 (MUC7)	Reproductive tract epithelial marker, modulates immune responses	Epithelial cell lines^[93,94]^
	MicroRNA 34 family (*MIR34*)	Tumor suppressor	Colorectal cancer cells^[95]^
	MicroRNA 200 family (*MIR200*)	EMT and differentiation regulator	mdck ^[96,98]^
	Occludin (*OCLN*)	Tight junction component, integral membrane protein	Mouse cultured epithelial cell lines^[86]^
	Phosphatidylethanolamine binding protein 1 (*PEBP1*)	Metastasis suppressor	Metastatic prostate cancer cells^[99]^
	Phosphofructokinase, platelet (*PFKP*)	Metabolic reprogramming	Breast cancer, MCF-7^[100]^
	Phospholipase D (*PLD*)	Chemotaxis, proliferation, cell signaling	Human breast cancer cell lines^[101]^
	Phosphatase and tensin homolog *(PTEN)*	Tumor suppressor	mdck ^[102]^
	Snail family transcriptional repressor 2 (*SNAI2*)	Promotes EMT and cell migration	Ovarian cancer^[103]^
	Vitamin D receptor (*VDR*)	Proinflammatory; inhibits EMT	Human colon cancer^[104]^
**Transcriptional Activation**	Collagen type I alpha 1 chain (*COL1A1*)	Collagen structural component	Hepatocellular carcinoma^[105]^
	C-X-C motif chemokine ligand 1/2 (*CXCL1/2*)	Recruitment of immunosuppressive cells	Hepatocellular carcinoma^[106]^
	Fibronectin 1 (*FN1*)	Cell adhesion, differentiation, and migration	Hepatocellular carcinoma, human carcinoma cell lines/epithelial cells^[31,105]^
	Matrix metallopeptidase 9 (MMP9)	Degradation of ECM	mdck ^[107]^
	Prostaglandin-endoperoxide synthase 2 (*PTGS2*)	Inflammatory pathway mediator	Hepatocellular carcinoma^[105]^
